# Substrates and oxygen dependent citric acid production by *Yarrowia lipolytica*: insights through transcriptome and fluxome analyses

**DOI:** 10.1186/s12934-017-0690-0

**Published:** 2017-05-08

**Authors:** Wael Sabra, Rajesh Reddy Bommareddy, Garima Maheshwari, Seraphim Papanikolaou, An-Ping Zeng

**Affiliations:** 1 Institute of Bioprocess and Biosystems Engineering, Hamburg University of Technology, Denickestrasse 15, 21071 Hamburg, Germany; 2 Department of Food Science and Human Nutrition, Agricultural University of Athens, 11855 Athens, Greece; 3 Synthetic Biology Research Centre, University of Nottingham, Nottingham, NG7 2RD UK

**Keywords:** Citric acid, Dual substrate, Oleaginous yeast, Pentose phosphate pathway, Glyoxylate cycle

## Abstract

**Background:**

Unlike the well-studied backer yeast where catabolite repression represents a burden for mixed substrate fermentation, *Yarrowia lipolytica*, an oleaginous yeast, is recognized for its potential to produce single cell oils and citric acid from different feedstocks. These versatilities of *Y. lipolytica* with regards to substrate utilization make it an attractive host for biorefinery application. However, to develop a commercial process for the production of citric acid by *Y. lipolytica*, it is necessary to better understand the primary metabolism and its regulation, especially for growth on mixed substrate.

**Results:**

Controlling the dissolved oxygen concentration (pO_2_) in *Y. lipolytica* cultures enhanced citric acid production significantly in cultures grown on glucose in mono- or dual substrate fermentations, whereas with glycerol as mono-substrate no significant effect of pO_2_ was found on citrate production. Growth on mixed substrate with glucose and glycerol revealed a relative preference of glycerol utilization by *Y. lipolytica*. Under optimized conditions with pO_2_ control, the citric acid titer on glucose in mono- or in dual substrate cultures was 55 and 50 g/L (with productivity of 0.6 g/L*h in both cultures), respectively, compared to a maximum of 18 g/L (0.2 g/L*h) with glycerol in monosubstrate culture. Additionally, in dual substrate fermentation, glycerol limitation was found to trigger citrate consumption despite the presence of enough glucose in pO_2_-limited culture. The metabolic behavior of this yeast on different substrates was investigated at transcriptomic and ^13^C-based fluxomics levels.

**Conclusion:**

Upregulation of most of the genes of the pentose phosphate pathway was found in cultures with highest citrate production with glucose in mono- or in dual substrate fermentation with pO_2_ control. The activation of the glyoxylate cycle in the oxygen limited cultures and the imbalance caused by glycerol limitation might be the reason for the re-consumption of citrate in dual substrate fermentations. This study provides interesting targets for metabolic engineering of this industrial yeast.

**Electronic supplementary material:**

The online version of this article (doi:10.1186/s12934-017-0690-0) contains supplementary material, which is available to authorized users.

## Background

With increasing focus on the development of sustainable technologies, the necessity for converting alternative, cheaper and waste carbon sources is becoming obvious. A further and equally important consideration is the development of sustainable microbial platforms to produce a variety of products from a vast array of biomass. Raw glycerol, which has become available in large quantities, and glucose, the most abundant carbon source in nature are applied in different industrial scale bioprocesses. Indeed, and with respect to price fluctuation and availability, finding efficient cell factories for the simultaneous conversion of these substrates to value added products will offer greater flexibility*. Yarrowia lipolytica*, an oleaginous yeast, is one of the most extensively studied ‘‘non-conventional’’ yeasts. Being a strictly aerobic microorganism it is capable of producing important metabolites and accumulates large amount of lipids from variety of substrates [1–4]. *Y. lipolytica* is known for its ability to secrete organic acids including tricarboxylic acid (TCA) cycle intermediates or precursors such as succinic, acetic, citric and isocitric acids [5–8]. Availability of genome sequence and the existence of suitable tools for genetic manipulation have made it possible to use the metabolic function of this species for biotechnological applications [9, 10]. One of the most striking features of this yeast is the presence of several multigene families involved in these metabolic pathways. The complexity and multiplicity of these genes enable *Y. lipolytica* to use and valorize a wide range of raw substrates and wastes [11–16].

It is well documented that in various yeast strains including *Y. lipolytica*, nitrogen limitation gradually change the metabolism from cell growth phase to citric acid production and/or storage lipid accumulation phase [17]. Intracellular citrate is known to be the prime carbon source for fatty acid synthesis, and can also regulates glucose metabolism via its allosteric inhibition of phosphofructokinase [7, 18, 19]. Citrate is a key intermediate in both catabolism and anabolism, and it occupies a prominent position in the yeast energy metabolism, and therefore, its production is affected by the cultivation conditions and the energy and C-source used. Despite an increased number of biotechnological applications performed by several wild or genetically engineered *Y. lipolytica* strains [13–15, 20–22] relatively few studies have focused on the metabolism, growth kinetics and product formation in mixed substrate fermentation [7, 23]. In general, dual substrate fermentation by oleaginous yeasts has been little investigated [24, 25]. Hen*ce,* the starting point of this work is to compare the growth of *Y. lipolytica* and products formation in dual substrate of glucose and glycerol versus mono-substrate fermentation. Since growth and citric acid production are all known to be affected by oxygen availability, mono- and dual substrate fermentations were carried out in cultures with or without control of dissolved oxygen tension (pO_2_). Here, we report that controlling the pO_2_ at 50% air saturation enhance citric acid production when glucose was present in mono- or in dual substrate fermentation, whereas, no significant effect of the pO_2_ on citric acid production on glycerol was observed. Moreover, utilization of citric acid despite the presence of excess glucose in the medium was observed after glycerol limitation in the pO_2_ uncontrolled cultures. In order to understand such metabolic behavior, RNA-seq based transcriptome and ^13^C-based metabolic flux analyses were performed.

## Results and discussion

### Glycerol as a favored substrate for growth in the dual substrate fermentation in shake flask cultures

Mono-versus dual substrate fermentations were first investigated in shake flask cultures with an initial carbon source concentration of 50 g/L. As shown in Table 1, in mono-substrate fermentation the biomass production increased slightly with glycerol (8 g/L) compared to glucose (7.1 g/L) as the sole C-source. In the dual substrate fermentation, a much higher consumption rate of glycerol against glucose was observed. Glycerol in general, was much more rapidly and efficiently assimilated by *Y. lipolytica* compared with glucose [7, 13, 23]. The lipid accumulation peaked at relative early stage in the growth phase in all flask cultures and remained constant or even decreased. Citric acid production increased during the later stages of cultivation which coincided with the depletion of lipids. Similar to previous reports, the re-consumption of citric acid during the course of cultivation in shake flasks was not observed [26, 27]. On the other hand, the total biomass concentration slightly increased at the later fermentation stages, suggesting the synthesis of cellular compounds other than lipids (i.e. polysaccharides or peptides [28, 29]). Low-molecular weight sugar alcohols like mannitol and erythritol was also reported to be produced by different strains of *Y. lipolytica* [12, 21, 22, 30–33]. Fatty acid (FA) compositions of the cellular lipids performed with mono-substrate fermentations revealed no significant modifications. The main FAs irrespective of the time and the substrate employed were oleic acid (^Δ9^C18:1; concentration ca. 43 ± 3% w/w of total lipids), palmitic acid (C16:0; concentration ca. 16 ± 3% w/w of total lipids), linoleic acid (^Δ9,12^C18:2; concentration ca. 17 ± 3% w/w of total lipids) and stearic acid (C18:0; concentration ca. 9 ± 3% w/w of total lipids). Citric acid secretion, on the other hand, was initiated after nitrogen limitation (observed at 30 ± 5 h after inoculation, data not shown) and was uninterruptedly secreted throughout the whole fermentation time. Its accumulation was comparable in all flasks with mono or dual substrates fermentation (see Table 1). Indeed, in such slightly acidic environment, an inhibitory action of citric acid on the metabolism of *Y. lipolytica* cannot be excluded. Therefore, the effect of substrate on the growth and product formation was further studied in controlled bioreactor.

**Table 1:** Biomass (X), lipid accumulation and citric acid production (Cit) of *Y. lipolytica* grown in nitrogen limited medium on glucose (Glc), glycerol (Glol) or blend of both

	Time (h)	Glol_0_ (g/L)	Glc_0_ (g/L)	Glol_cons_ (g/L)	Glc_0cons_ (g/L)	X (g/L)	Lipid (%, w/w)	Cit (g/L)	Y_cit/substrate_ (g/g)
a	25.5	48.1	–	9.0	–	5.1	14.5	0.6	0.07
b	161	48.1	–	47.9	–	8.0	4.1	21.8	0.45
a	29	–	50.9	–	11.4	4.2	11.9	1.1	0.10
b	185	–	50.9	–	50.2	7.1	5.2	21.0	0.42
a	43.5	31.1	18.6	13.2	2.3	4.0	11.1	2.7	0.17
b	230	31.1	18.6	31.1	18.6	7.5	3.3	22.9	0.46
a	24.5	18.9	33.0	9.9	1.5	4.5	14.0	0.9	0.08
b	235	18.9	33.0	18.9	31.9	7.1	7.1	21.1	0.42

Values are given for the two situations: (a) the maximum quantity of lipids in DCW was achieved; (b) the maximum quantity of citric acid was produced. Culture conditions: initial pH 6.5, pH ranging between 5.5 and 6.5, incubation temperature *T* = 28 ± 2 °C

### Oxygen requirements for citric acid production depends on the nature of the carbon source

**Fig. 1:**
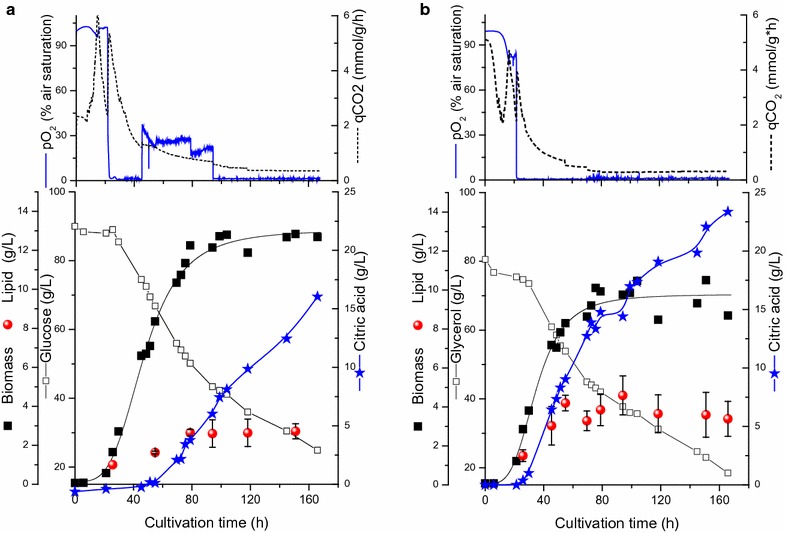
Biomass, products formation, CO_2_ production rate and substrate consumption in a pO_2_ uncontrolled batch culture of *Y. lipolytica* grown on glucose (**a**) or glycerol (**b**) as the sole carbon source

A set of cultivations was performed using either glucose or glycerol (Fig. 1a, b) as the sole carbon source, at an initial concentration of 90 g/L and without the control of dissolved oxygen. In general, *Y. lipolytica* grew faster on glycerol with a maximum specific growth rate of 0.15 h^−1^ compared to 0.13 h^−1^ with glucose as the sole carbon source. Although, different limitations stimulate lipid accumulation in oleaginous yeasts, nitrogen limitation has been used more extensively to create an environment suitable for its synthesis [12, 34]. Nitrogen limitation occurs at earlier stage during the cultivation (10–15 h) and a decrease in the specific CO_2_ production rate (in mmol/g/h, first peak) was observed (Fig. 1). Shortly after nitrogen limitation, dissolved oxygen concentration (pO_2_) became limited in both cultures (20–22 h) with the characteristic decrease in the ${\text{q}}_{{{\text{CO}}_{2} }}$ (second peak) and remained limited throughout the fermentation, especially on glycerol (Fig. 1). Lipid contents of *Y. lipolytica* cells (as % of dry weight) increased significantly with glycerol as the carbon source and reached 40% compared to 23% with glucose. Indeed, the requirement of glycerol as a direct precursor for triacylglycerides (TAG) might explain such results. Glycerol as favored C-source for lipid accumulation was also recently observed in *Rhodosporidium toruloides* [29, 35].

Interestingly, with glucose as the sole carbon source, four different phases can be seen. The first phase in which the cells grew in the lag and the early logarithmic phase with excess dissolved oxygen concentration. The second phase was characterized by increased oxygen consumption and CO_2_ production rate parallel to exponential growth and hence a pO_2_ limited phase. An initiation of citric acid production characterizes the third phase which was parallel to the abrupt increase in pO_2_ values, indicating a nutrient limitation, and finally the fourth phase which is a stationary growth phase with continued citrate production under pO_2_ limited conditions (Fig. 1). On the other hand, with glycerol as the sole carbon source, citric acid production was initiated at the onset of oxygen limitation. In both mono-substrate fermentations with the individual substrate, the citrate production was initiated at the mid logarithmic phase and continued with the onset of the stationary phase (Fig. 1). Previously, Morgunov et al. [19] reported that after nitrogen limitation and at the onset of stationary phase in *Y. lipolytica*, the activity of citrate synthase remained high, whereas the activities of citric acid degrading enzymes such as aconitate hydratase and NAD-dependant isocitrate dehydrogenase were considerably decreased. Since oxygen availability will affect most of the TCA cycle enzymes, the aim of the following part was to compare the growth and citric acid production using mono-substrate fermentation in pO_2_ controlled bioreactors.

**Fig. 2:**
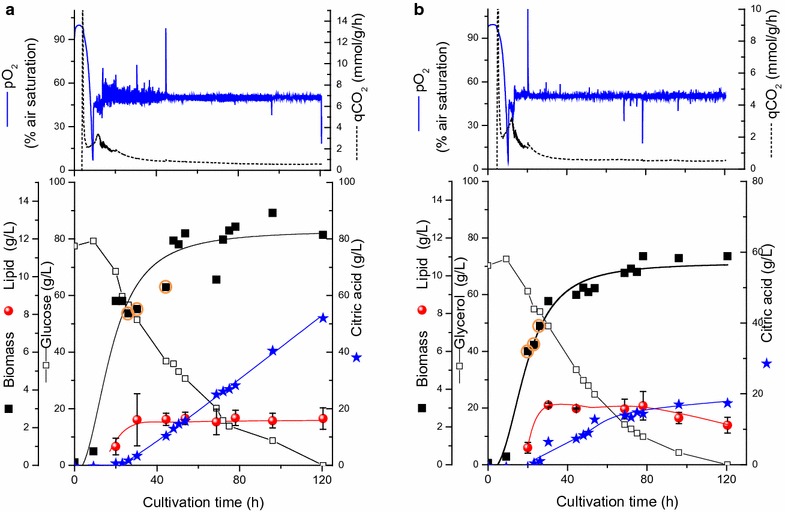
Time profiles of cell growth, lipid, citric acid production, CO_2_ production and substrate utilisation of *Y. lipolytica* cells grown at controlled pO_2_ of 50% air saturation with glucose (**a**) or glycerol (**b**). Samples taken for ^13^C flux analysis were marked

As seen in Fig. 2a, b and Table 2, the specific growth rates increased in pO_2_ controlled cultivation with either glucose or glycerol as substrate. The lipid accumulation, on the other hand, decreased in both the cultivations compared to the oxygen uncontrolled cultivation (Table 2). The same behavior of lipid accumulation was obtained previously by Papanikolaou and Aggelis [36].

**Table 2:** The effect of controlling the dissolved oxygen tension (pO_2_) on biomass, lipid and citric acid production by *Y. lipolytica* grown in nitrogen limited medium with glucose, glycerol or blend of both

Substrate	µ (h^−1^)	Biomass	Lipid	Citric	Citric acid yield in g/g substrate	
	Log-phase	(g/L)	(g/L)	(g/L)	(Maximum) in stationary phase	overall
pO_2_ uncontrolled						
Glucose	0.13	12.8	3	16.1	0.44	0.23
Glycerol	0.15	9.03	3.6	23.4	0.39	0.39
Blend	0.16	12.3	4.25	Varied	0.35	0.29
Blend with BH	0.11	14.2	2.4	Varied	0.41	0.27
pO_2_ controlled						
Glucose	0.2	12.2	2.4	55.0	0.83	0.58
Glycerol	0.21	11.03	2.1	17.8	0.3	0.27
Blend	0.22	11.9	2.4	50.1	0.76	0.6
Blend with BH	0.18	16.7	2.5	53.5	0.8	0.6

During growth of *Y. lipolytica* strains under nitrogen-limited condition, three types of lipid metabolism have been presented in literatures. The typical “oleaginous” metabolism; in which lipid accumulation is triggered after nitrogen limitation in significant quantities, while low or negligible quantities of extra-cellular metabolites are simultaneously produced (mostly citric acid and to lesser extent polyols [37]). An atypical “oleaginous” metabolism; in which lipid is initially stored after nitrogen limitation, after which lipid accumulation decreases while citric acid production occurs uninterruptedly [15]. A third atypical “oleaginous” metabolism was also described in which lipid accumulation starts at slow rates inside the yeast cells without degradation and with citric acid being constantly secreted extracellularly [30]. By taking into consideration the results achieved with the strain used in the current investigation, it can be considered that ACA-DC 50109 follows the second or the third category of metabolism.

In fact, the most significant changes were recorded in the citrate production by *Y. lipolytica* on both substrates. Oxygen stimulated citrate production only if glucose was used as the C-source (average citric acid titers were 55 and 16 g/L at pO_2_ controlled and uncontrolled, respectively) whereas no significant change of citrate production was detected on glycerol (18 and 23 g/L at pO_2_ controlled and uncontrolled, respectively, Table 2). Previously, Kamzolova et al. [5] reported that oxygen requirements for growth and citric acid synthesis were found to depend on the iron concentration of the medium. Only at lower iron concentration (0.7 mg/L) higher oxygenation was reported to enhance citric acid production in *Y. lipolytica* with glycerol [5]. This in fact can explain the results obtained with glycerol, where no significant differences in citric acid production was obtained with and without pO_2_ control in our iron rich medium (150 mg/L) (Fig. 1). In general, the difference in the nature of metabolism of both glycerol, an energy-poor carbon source and glucose and their effect on acid production are still not understood and deserve more in depth studies.

### Higher fluxes to pentose phosphate pathway (PPP) and lower to TCA cycle were recorded with cells grown on glucose compared to glycerol at pO_2_-controlled cultures

**Fig. 3:**
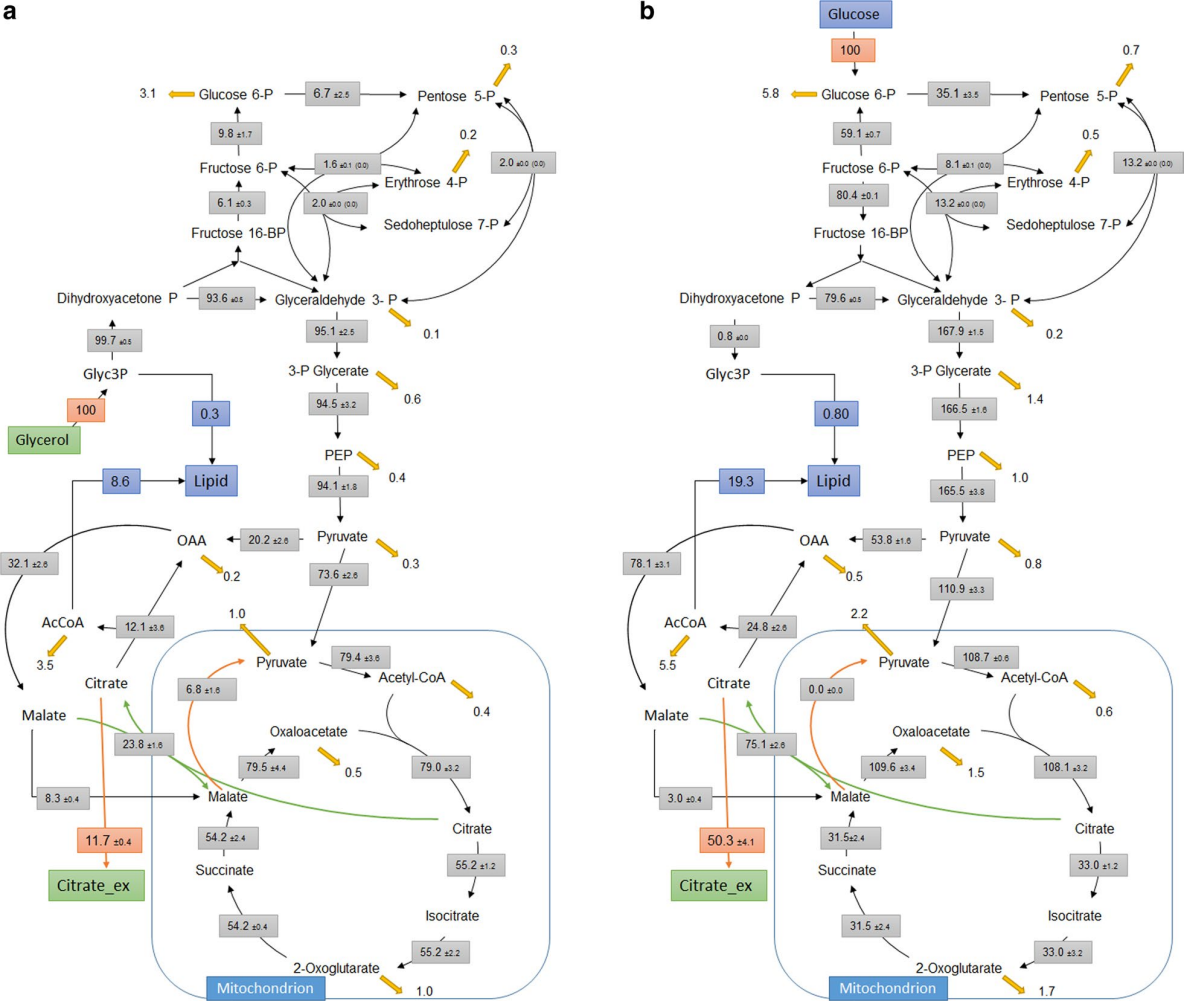
Flux distribution of *Y. lipolytica* grown on glycerol (**a**) or glucose (**b**) as the sole carbon source under O_2_ controlled conditions. Values are in mmol g_DCW_^−1^ h^−1^ normalized to C-source uptake rate which is set as 100%. *Arrows* indicated flux towards biomass precursors

To gain more insights into the intracellular fluxes, ^13^C metabolic flux analysis were done with *Y. lipolytica* grown under pO_2_ controlled conditions with either glucose or glycerol as the sole C-source (Fig. 3). Biomass samples for the ^13^C flux analysis were taken during the exponential growth phases of both cultures. The most significant changes were found in the relative fluxes through the phosphate pentose pathway (PPP), TCA cycle and citrate production. With glycerol as the sole carbon source, only 6.7% of its uptake rate was directed to the PPP compared to 35% with glucose. On the other hand, higher fluxes toward TCA cycle were observed with glycerol rather than glucose as mono-substrate (Fig. 3). The relatively lower TCA cycle and higher PPP fluxes could explain the higher citrate produced with glucose as the sole carbon source. The higher PPP fluxes could be a consequence of higher NADPH requirement on glucose needed for growth and biosynthesis (Table 2). Moreover, the higher PPP fluxes would also reduce the fluxes toward the NADP dependent isocitrate dehydrogenase, reported to be present in *Y. lipolytica*, the major citrate degrading enzyme [38]. On the other hand, the relative higher fluxes from glycerol 3-phosphate to dihydroxyacetone phosphate via the membrane bound glycerol-3-phosphate dehydrogenase with a concurrent reduction of flavin adenine dinucleotide (FAD) might explain the relatively higher TCA cycle fluxes on glycerol. The produced FADH_2_ is known to pass their electrons on to ubiquinone found in the inner membrane of the mitochondria and ultimately to oxygen [39].

### Citrate re-consumption after glycerol limitation in the oxygen limited dual substrate culture

**Fig. 4:**
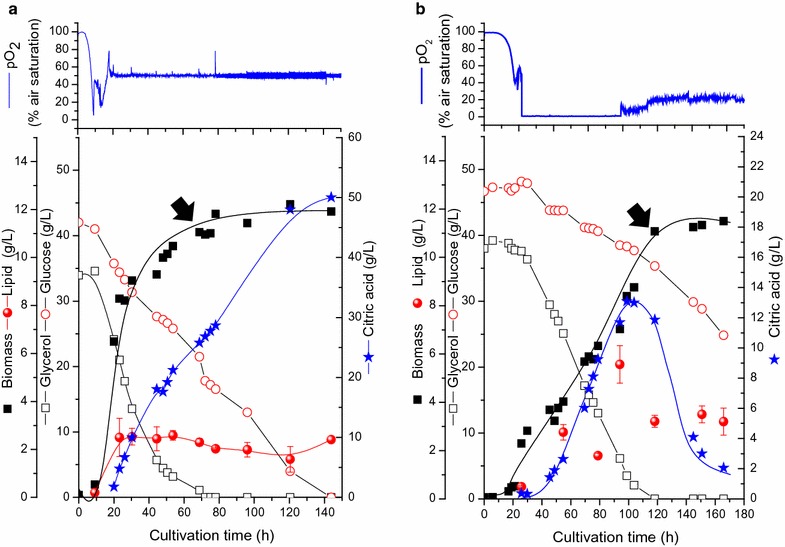
Biomass, products formation, and substrate consumption in a pO_2_ controlled (**a**) and uncontrolled (**b**) culture of *Y. lipolytica* grown on a mixture of glycerol and glucose. *Arrows* show the onset of glycerol limitation

The effect of controlling the dissolved oxygen tension on the growth and product formation by *Y. lipolytica* with dual-substrate utilization was also examined. Both substrates were simultaneously consumed but with different rates, and these rates increased at pO_2_ controlled conditions. Glycerol was consumed at 0.7 and 0.46 g/L*h, whereas glucose consumption rates were 0.3 and 0.15 g/L*h at pO_2_ controlled and uncontrolled conditions, respectively (Fig. 4). The higher substrate consumption rates obtained relatively with pO_2_ controlled conditions were reflected by the increase in the specific growth rate from 0.16 to 0.22 h^−1^ at pO_2_ uncontrolled and controlled conditions.

As shown in Fig. 4, after glycerol limitation, and despite the presence of excess glucose, utilization of citrate was observed in the pO_2_ uncontrolled culture. In the same culture, growth arrest was also observed after glycerol limitation (Fig. 4b). The same trend was also noticed using blend with spruce biomass hydrolysate in pO_2_ uncontrolled batch cultures (Additional file 1: Figure S1). Oxygen limitation in mono-substrate cultivation on either glucose or glycerol as shown in Fig. 1 didn’t trigger citrate re-consumption (with the current strain, or with *Y. lipolytica* A101 as reported recently [16]). This physiological behavior in dual-substrate fermentation is reported here for the first time. *Y. lipolytica* was reported to degrade citric acid after C-source limitation in mono-substrate fermentation [7], but was never investigated in dual substrate fermentation and especially when a carbon source like glucose was in excess. On the other hand, at controlled pO_2_ conditions, the yeast continued to produce citric acid as the major product, even after the assimilation of the favorable carbon source (glycerol). The citric acid yield was very similar to the mono-substrate fermentation in presence of glucose at pO_2_ controlled conditions (0.6 g/g _substrate_, Table 1). To understand the behavior difference in *Y. lipolytica* metabolism in dual substrate fermentation at oxygen limited and excess conditions, detailed transcriptomic analysis were done.

**Fig. 5:**
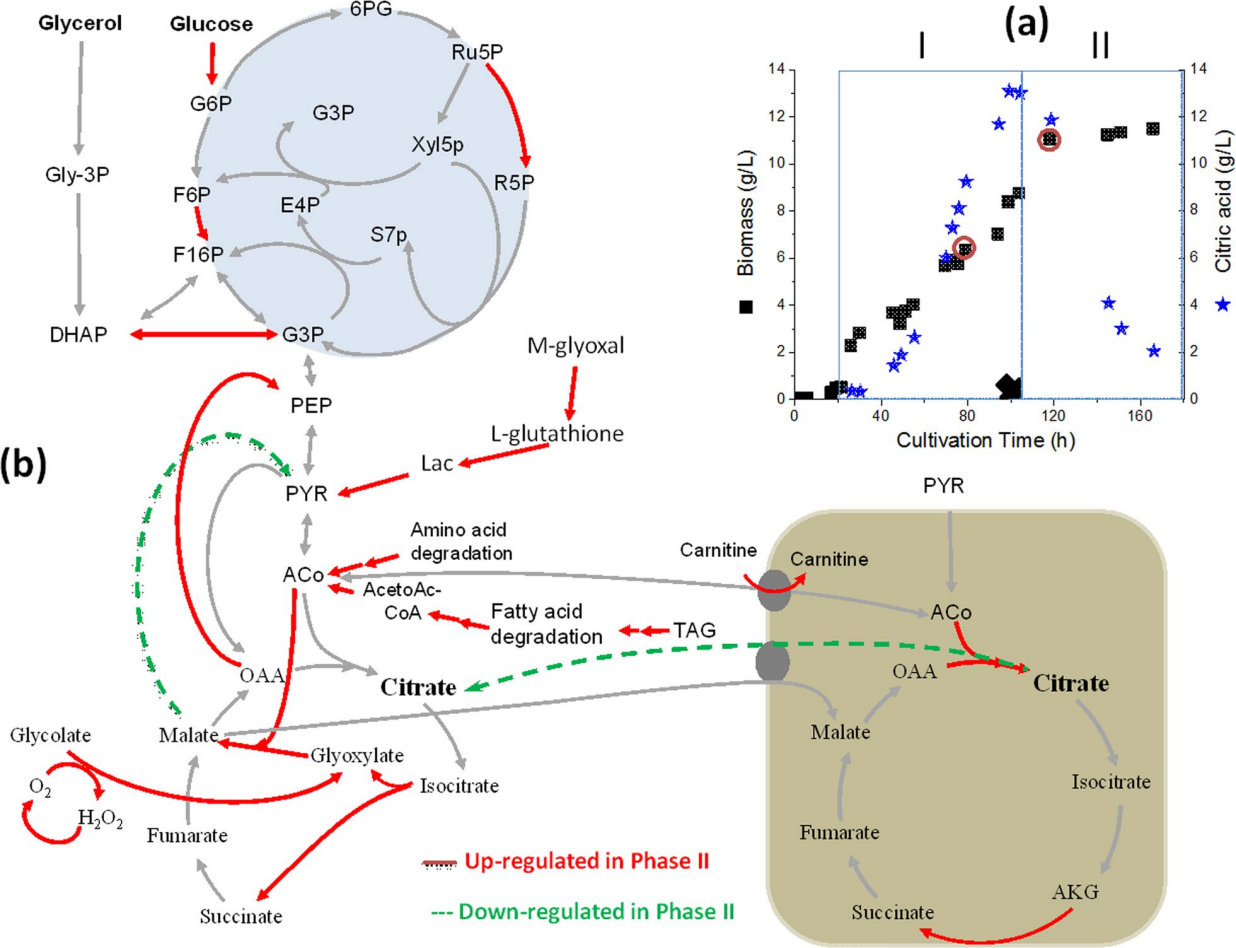
Time points of sampling for the transcriptomic analysis of *Y. lipolytica* cells grown at pO_2_ uncontrolled conditions before and after glycerol limitation (**a**), and results from the transcriptomic analysis showing the upregulated genes and their enzymatic reactions colored in *red solid lines* and the downregulated in *green dash lines* (**b**)

**Fig. 6:**
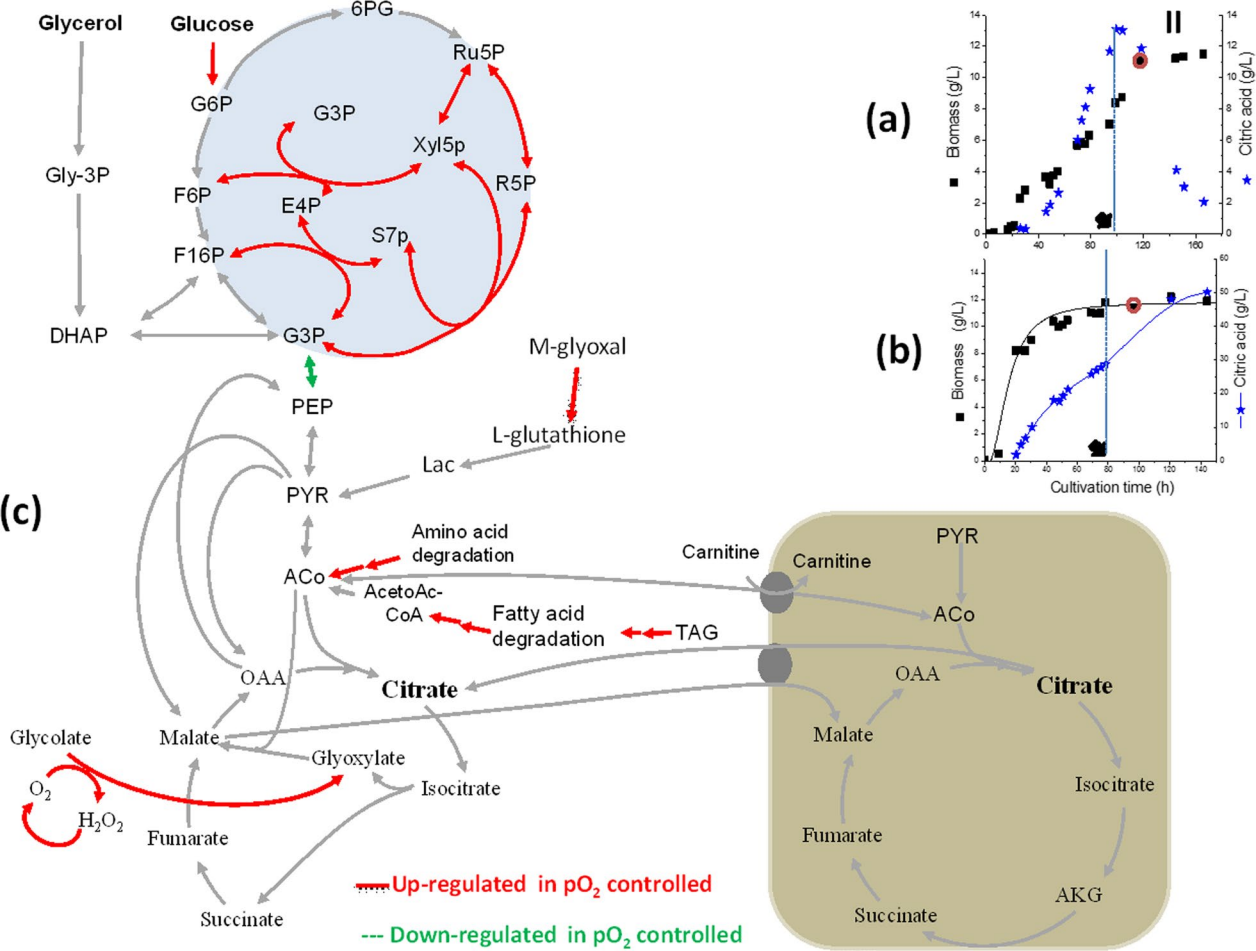
Time points of sampling for the transcriptomic analysis of *Y. lipolytica* cells grown at pO_2_ uncontrolled (**a**) and controlled conditions (**b**) after glycerol limitation (*arrow*), and results from the transcriptomic analysis showing the upregulated genes and their enzymatic reactions colored in *red solid lines* and the downregulated in *green dash lines* (**c**)

With either pO_2_ controlled or uncontrolled conditions, samples were collected before (phase I) and after (phase II) glycerol limitation and two biological replicates for each phase were used for our analysis (Figs. 5, 6). Over 3 million pair-end sequence reads were obtained for each sample. These sequenced reads had >85% mapping rate to *Y. lipolytica CLIB122 GCA_000002525.1* genome assembly. Comparing between the pO_2_ controlled cultivation samples, of the 2205 genes that were expressed, about 640 and 290 genes were significantly up- and down-regulated after glycerol limitation (Additional file 1: Table 1). However, in the fermentation with pO_2_ uncontrolled where metabolic shift was noticed after glycerol limitation, almost 3000 genes were expressed; from which 946 and 367 genes were significantly up- and down-regulated (Additional file 1: Table 1) when compared between the sample. In our experiments a fold difference of minimum two between the two phases [Log_2_ (FPKM Phase II/FPKM Phase I)] was taken to be significant. Moreover, to classify the predicted functions of the transcripts, GO terms were assigned using Blast2GO (Additional file 1: Figures S2–S3).

### General stress metabolism after glycerol limitation in dual substrate fermentation with *Y.lipolytica*

Yeast cells are known to initiate a common gene expression program that generally protects the cell under stress [40]. This response, referred to as the environmental stress response, includes ~900 genes whose expression is stereotypically altered when yeast cells are shifted to stressful environments [40]. Irrespective of the pO_2_ control, several stress genes were found to be highly regulated after glycerol consumption. Interestingly, glycerol assimilating enzymes, namely glycerol kinase encoded by GUT1 (YALI0_F00484 g) and glycerol 3P dehydrogenase encoded by GUT2 (YALI0_B13970 g), were not significantly expressed (NSE) after glycerol limitation at both controlled and uncontrolled pO_2_ conditions (<2-fold change). Stress responses initiated by nitrogen limitation can be neglected as all samples for transcriptomic analysis were collected after nitrogen limitation. Examples are genes normally upregulated in response to osmotic stress (YALI0_F01210 g, 6.6-fold and YALI0_C00759 g, 2.3-fold at pO_2_-uncontrolled; YALI0_F01210 g 2.9 at pO_2_-controlled); starvation (YALI0_E31757 g, 5.2-fold at pO_2_-uncontrolled; YALI0_F14377 g, 2.9, YALI0_E20163 g, 2.6-fold at pO_2_-controlled), cell aging (YALI0_F31977 g, 2.4-fold at pO_2_-uncontrolled), oxidative stress genes (YALI0_E34265 g, 2.3-fold at pO_2_-uncontrolled), or chaperonin and other cellular stimuli (YALI0_C04884 g, 4.3-fold; YALI0_F25905 g, 2.4-fold at pO_2_-uncontrolled, YALI0_F00880 g, 2.4-fold at pO_2_-controlled) (Additional file 1: Table 1).

Growth arrest after glycerol limitation and irrespective to the pO_2_ control was also seen form the down-regulation of several growth related genes. Examples are some cell division control proteins that were downregulated in both cultures after glycerol limitation (CDC15:YALI0_F08165 g, −2.9 and −3.4-fold; CDC6:YALI0C00671 g, −4.6-fold and NSE; cell division cycle 20: YALI0_C03377 g, −2.1 and −2.4-fold; cell division cycle 14: YALI0_E16038 g, NSE and -2.4-fold at pO_2_-controlled and uncontrolled conditions, respectively, Additional file 1: Table 1). Growth cessation triggered by the limitation of the favorable substrate was previously investigated in *Clostridium pasteurianum* in a blend of glucose and glycerol [41].

Moreover, a large number of transport proteins, including multiple oligopeptide transporters were highly induced after glycerol limitation especially with pO_2_-uncontrolled conditions (YALI0_A00110 g, 11.9-fold and YALI0_D23485 g, 8.4-fold; YALI0_E19294 g, 6.9-fold; YALI0_E04510 g, 4.7-fold; YALI0_F30987 g, 4.3-fold; YALI0_B02398 g, 4.2-fold; YALI0_C18491 g, 4.0-fold; YALI0_C22616 g, 3.2-fold; at pO_2_-uncontrolled compared to, YALI0_D23485 g, 3.1-fold; YALI0_F18964 g, −5.0-fold; YALI0_C18491 g, −3.1-fold and YALI0_D19558 g, −2.4-fold; at pO_2_-controlled conditions). This may suggest that *Y. lipolytica* may use small peptides as nutrients, and such a behavior could support the growth of this yeast in natural environments where *Yarrowia* is naturally found (cheeses, yoghurts and meat). Similar behavior was recorded in *Candida albicans* when cells shifted from glucose, the preferred C-source to glycerol containing medium [42].

Allantoate permease was also highly regulated after glycerol limitation (relatively more at pO_2_-uncontrolled conditions, YALI0_A03091 g, 11.4-fold; YALI0_F28193 g, 10.8-fold; YALI0_F16423 g, 7.2-fold; YALI0_D13970 g, 4.5-fold; YALI0_C08569 g, 4.4-fold; YALI0_A00869 g, 3.8-fold; YALI0_C13574 g, 3.8-fold; YALI0_D05819 g, 2.7-fold; YALI0_C16907 g, 2.2-fold, compared to YALI0_A03091 g, 4.7-fold; YALI0_D13970 g, 4.2-fold; YALI0D13970 g, 3.8-fold; YALI0_C08569 g, 3.2-fold, YALI0_D13970 g, 2.4-fold at pO_2_-controlled experiment). Similarly, the gene YALI0_C07590 g for organic acid: sodium symporter activity was highly regulated after glycerol limitation especially in pO_2_-uncontrolled conditions (6.3-fold compared to 2.9 at pO_2_ controlled conditions*).* All these genes are related to RTG (retrograde signaling pathways) which are activated during a deficient mitochondrial function or in response to deficiencies in respiration.

### Upregulation of the glyoxylate cycle after glycerol limitation in the pO_2_-uncontrolled culture

Important genes associated with citric acid and central carbon metabolism in *Y. lipolytica* were studied in culture without pO_2_ control before and after glycerol limitation (Fig. 5). Although the glyoxylate and TCA cycles share common reactions, it is only the genes for the glyoxylate cycle that were upregulated after glycerol consumption in the pO_2_ un-controlled cultures (malate synthase: YALI0_D19140 g, 4.5-fold and YALI0_E15708 g, 5.4-fold, Fig. 5). Moreover, enzymes for precursors synthesis needed for gyloxylate cycle were found to be up-regulated. In addition to the glycolate oxidase for the synthesis of glyoxylate (YALI0_D12661 g, 2.2-fold), several genes for the replenishment of acetyl CoA were found to be up-regulated after glycerol consumption. Examples are several genes involved in fatty acid degradation (YALI0_C23859 g, 3.9-fold; YALI0_D24750 g, 5.6-fold; YALI0_F10857 g, 4.5-fold; YALI0_B10406 g, 3.5; YALI0_E11099 g, 3.8-fold), and amino acid degradation (especially leucine, valine and isoleucine, YALI0_D08690 g, 6.6-fold; YALI0_D23815 g, 5.2-fold, YALI0_B10406 g, 3.5-fold; YALI0_B22550 g, 2.3-fold; YALI0_E11099 g, 3.8-fold). Also several genes involved in peroxisome biogenesis, a compartment of central role in glyoxylate cycle function, were found to be up-regulated in the glycerol limited phase (YALI0_B22660 g, 4.2-fold; YALI0_A20944 g, 3.5-fold; YALI0C18689 g, 2.8-fold; YALI0_F28457 g, 2.8-fold; YALI0_E09405 g, 2.8-fold; YALI0_C05775 g, 2.2-fold; YALI0D26642 g, 2.1; YALI0_C09504 g, 2.1-fold). In control experiments without the metabolic shift with pO_2_ controlled condition, neither genes expression of glyoxylate cycle nor peroxisome biogenesis were changed significantly in response to glycerol consumption (Fig. 4a, Additional file 1: Figure S4).

The glyoxylate cycle is known to serve as a link between catabolic activities and biosynthetic capacities and enables cells to utilize fatty acids or C2-units as sole carbon source [43]. Unlike *S. cerevisiae*, *Y. lipolytica* prefers glycerol as C-source and quantitative studies on dual substrate fermentation with glucose are still very few [7, 23]. Therefore, known metabolic models for the traditionally applied yeast cell factories can hardly be used to explain the metabolic shift observed in *Y. lipolytica* in response to glycerol and oxygen limitation (Figs. 4b, 5). Definitely, oxygen limitation certainly affects the main function of mitochondria and energy generation by oxidative phosphorylation, and as shown in transcriptomic data (Additional file 1: Table 1), the four isozymes of the NADH dehydrogenases were upregulated after glycerol limitation at the pO_2_-controlled condition (YALI0_C17853 g, 4.3-fold and YALI0_C22319 g, 2.6, YALI0_C22319 g, 2.59-fold at pO_2_ controlled conditions) while all of these genes were found to be non-significantly expressed (NSE) under oxygen limited conditions. It seems likely that, under reduced function of mitochondria as a result of oxygen limitation, citrate which is produced in the mitochondria serves predominantly as a key substrate for energy generation (Fig. 5). Citrate exhaustion in the cytoplasm may ultimately trigger its re-consumption from the medium through organic acid transporters. Recently, Guo et al. [44] identified 6 putative genes responsible for the transport of organic acids in *Y. lipolytica*, from which only 5 genes encode proteins that can transport citrate. Form these 5 genes, only 3 genes were differentially expressed: YALI0_B19470 g (−4.5 and 2.3-folds), YLI0_C15488 g (3.3 and 4.3-folds), and YALI0E32901 g (NSE and 7.73-folds at pO_2_ controlled and uncontrolled conditions; respectively). Still it is not known whether efflux and influx of citrate are shared by the same proteins.

### Upregulation of the non-oxidative PPP is correlated to high citric acid production in *Y. lipolytica*

Higher citric acid production by *Y. lipolytica* was found to be dependent on the carbon source and oxygen availability (Figs. 2, 3). The high citrate production with glucose in monosubstrate cultivation (Fig. 2) coincide with the relatively higher fluxes toward PPP than with glycerol as sole C-source (Fig. 3). Interestingly, when the glycerol limited phases at both pO_2_ controlled and uncontrolled conditions were compared, up-regulation of almost all the genes of the non-oxidative PPP were observed in the pO_2_ controlled culture compared to the pO_2_ uncontrolled one (Fig. 6). In fact, in comparison to glycerol and similar to the glucose as monosubstrates, the PPP fluxes were also significantly higher on dual substrate fermentation with pO_2_ control (Additional file 1: Figure S5). The role of PPP on the metabolism and citric acid production in *Y. lipolytica* is still not clear and deserve more studies. Whereas an oxidative PPP was shown to be essential for lipid production from glucose in *Y. lipolytica* [45], our results showed that the upregulation of the non-oxidative PPP genes could lead to high citrate production from glucose (Fig. 6). Essentially the glycolytic pathway is common to all yeast species and the carbon flux regulation is being done at the level of the pentose phosphate pathway. Whether glucose 6-phosphate enters glycolysis or the pentose phosphate pathway depends on the current needs of the cell and on the concentration of NADP+ in the cytosol. Previously, it was reported that the marked effect of the NADP+ level on the rate of the oxidative PPP ensures that NADPH generation is tightly coupled to its utilization in reductive biosynthesis such as lipid accumulation, whereas, the nonoxidative phase of the PPP is controlled primarily by the availability of substrates [46].

*Yarrowia lipolytica* is known for both efficient citrate excretion and high lipid productivity under stress conditions such as nitrogen limitation. Under N-limitation, the prevalence of oxygen limitation was shown to enhance lipid production while decrease citrate production on glucose significantly (Table 2 and [47]). Besides the complex interplay between the oxygen effect and the nature of substrate in regulating citrate production, the availability of the C-source and the efficiency of the glycolytic flux were recently found to have a significant impact on *Y. lipolytica* metabolism [47]. Decreasing the glycolytic flux through glucose uptake rate in controlled fed batch culture was reported to decrease citrate production and enhanced lipid production by this yeast [47]. In addition to the double limitations exerted by N- and O_2_ in *Y. lipolytica* cells grown in pO_2_ uncontrolled condition, the onset of an additional glycerol limitation shifted the metabolism significantly (Fig. 4b), and the resulted low glycolytic fluxes triggered a decreased citric acid production and consequently re-consumption of the medium. Indeed, the different in the fluxes toward PPP with the two substrates (Fig. 3) also played here a significant role. As *Y. lipolytica* consumes both these substrates simultaneously with preference to glycerol, it looks like that initially glucose is only used to generate the PP flux for NADPH, whereas glycerol mainly is driven towards lipid and lower glycolysis. Hence, at the moment of glycerol limitation, the cells fail to redirect glucose through the lower glycolysis and TCA cycle rather, they use citric acid as the preferred carbon source. For the development of *Y. lipolytica* as a microbial cell factory for citric acid production and other biotechnological applications [48], the differences between the fluxes with respect to the activity of the PPP, TCA cycle and glyoxylate cycle had to be optimized and understood especially with dual substrates.

## Conclusion

Citric acid production by *Y. lipolytica* grown on either glucose, glycerol, or mixture of them was investigated in cultures with controlled or limited pO_2_. The different nature of the two substrates, especially their reduction degree was reflected by the different fluxes toward PPP and TCA cycle and finally citrate as evidenced by ^13^C analysis for mono-substrate fermentations. Relatively higher PPP and lower TCA cycle fluxes were recorded in *Y. lipolytica* cells grown under sufficient oxygen supply on glucose or on blend with glycerol but not in cells grown with glycerol as the sole C-source. Down-regulation of the TCA cycle fluxes triggered by oxygen limitation led to an increased flux to lipid formation, and less to citrate production. The TCA cycle fluxes were further decreased in oxygen limited—dual substrate fermentation after glycerol limitation. The imbalance exerted in the fluxes of PPP and TCA cycle as a result of glycerol limitation resulted in up-regulation of the glyoxylate pathway and the re-utilization of citrate despite the presence of excess glucose. This study highlights the importance of controlling the oxygen supply for citrate production by *Y. lipolytica* on mixed substrates.

## Methods

### Microorganism and media

*Yarrowia lipolytica* ACA DC 50109 was used in the present. The strain was maintained at −80 °C on potato dextrose medium with 20% glycerol (w/v). The medium for seed culture and batch fermentations are similar to that reported previously [7]. For isotope labeled experiment, yeast extract was replaced by vitamins solution which contained (per liter): biotin 0.05 g; p-amino benzoic acid 0.2 g; nicotinic acid 1 g; Ca-pantothenate 1 g; 19 pyridoxine–HCl 1 g and thiamine–HCl 1 g. The medium was supplemented with 30 g/L glucose or glycerol and for mixed substrate cultivations 1:1 mixture of glucose and glycerol were added. For the labelled experiments, 100% 1-^13^C glucose, 99% atom (Sigma, Germany) or 20% U-^13^C6 glucose 99% atom (EURISO-TOP GmbH, Germany) or 20% U-^13^C3 glycerol (99% atom, Cortecnet, France) were used. For mixed substrate labelled experiments, 50% U-^13^C3 glycerol, 20% 1-^13^C glucose and 30% naturally labelled glucose were used.

### Cultivations

Flask experiments were conducted in 250-mL non-baffled flasks, containing 50 mL of nitrogen limited growth medium. Flasks were shacked at 200 rpm in an orbital shaker (Lab-Line, USA) at 28 ± 1 °C. To minimize the drop of pH KH_2_PO_4_ and Na_2_HPO_4_ at initial concentrations of 12 g/L of each were added. The initial pH in these media was 6.4 ± 0.1, and effort was done to maintain the pH of the culture medium to values >5.0, by aseptically adding few drops of concentrated KOH solution (≈5 M). The initial carbon source concentrations used were 50 g/L in all flask experiments. Mono-substrate and dual substrate fermentation with different variations of glucose to glycerol concentration were also compared.

Batch fermentations were performed in Dasgip fermenters (Dasgip, Jülich, Germany). 1.5 L capacity vessels with an initial working volume of 500 mL were used. For ^13^C labelled experiments 200 mL capacity fermenter vessels with an initial working volume of 120 mL were used. One replicate with labeled glycerol (20% U-^13^C_3_) and two replicates with un-labeled glycerol were performed. In case of glucose as the substrate, one replicate with 100% 1-^13^C glucose and second replicate with 20% U-^13^C_6_ glucose and third replicate with unlabeled glucose were performed. In case of mixed substrates one set of experiment was performed with labeled substrates and the remaining two sets were performed with naturally labeled substrates. Agitation and temperature were maintained at 400 rpm and 30 °C respectively. pH was maintained at 6.0 by automatic addition of 5 M NaOH. Dissolved oxygen was maintained at 50% air saturation throughout the experiment by adjusting the oxygen concentration in the inlet gas. Aeration was set to 0.5 vvm. The off-gas from the fermenters was analyzed by the Dasgip off-gas analyzing system to determine the real time oxygen uptake and CO_2_ evolution rates.

## ^13^C labeled substrate experiments and metabolic flux estimation

For ^13^C-labeled experiments, cells from the precultures were washed prior to inoculation in order to reduce the effect of unlabeled biomass. The washed cells were used to inoculate the fermenters to an initial OD_600_ of 0.03. Media and initial substrate concentrations were similar to what was described before. Samples collected during the mid-exponential phase were treated and analyzed using GC–MS (Agilent GC 7890B coupled to a 5977 MSD) as described previously [49].

The compartmentalized metabolic network model of *Y. lipolytica* metabolism was constructed based on a previous model of *R. toruloides* [26]. Briefly, the metabolic network is comprised of glycolysis, PP pathway, TCA cycle, fatty acid and biomass synthesis. Citrate from mitochondria is transported to the cytosol through the citrate-malate shuttle. Acetyl-CoA in the cytosol is assumed to be formed only through the ATP: citrate lyase (ACL) reaction. Oxaloacetate (OAA) in the cytosol was assumed to be formed by the anaplerotic reaction, pyruvate carboxylase and the ACL. A cytosolic malate dehydrogenase (NAD^+^ forming) converting OAA to malate in the cytosol was included. Malate transport from cytosol to mitochondrial membrane via a dicarboxylate carrier was included. The software toolbox INCA [50] was used for parameter estimation and evaluation in Matlab 2013a.

### Extracellular metabolites and lipid analysis

Cells were harvested during the cultivations after centrifugation (5000 rpm, 10 min at 4 °C). Cell dry weight was determined gravimetrically after drying the harvested cells in an oven at 80 °C to a constant weight. Total lipid extraction with chloroform/methanol mixture (2:1 v/v) was performed as described in previous work [7]. Fatty acid composition was done as described previously by Papanikolaou et al. [34]. Lipid free biomass was calculated after subtracting the lipid content from the total biomass. Quantification of glucose, glycerol and organic acids was carried out using high-performance liquid chromatography (HPLC; Kontron Instruments, United Kingdom) with separation on an Aminex HPX-87H column at 60 °C with 0.005 M H_2_SO_4_ and detection via refractive index or by UV absorption at 210 nm. Ammonia concentration in the supernatant was determined by photometric measurements using a kit from Macherey–Nagel, Germany.

### RNA extraction and sequencing

For whole transcriptome sequencing, samples from the mixed substrate (1:1 mixture of glucose and glycerol) cultivations were withdrawn before and after glycerol limitation. Approximately 3 × 10^8^ cells were collected by centrifugation (5000 rpm, 2 **°**C for 5 min) and the biomass was immediately re-suspended in RNA*later* ^®^ Stabilization Solution (Life technologies, Ambion). The total RNA was extracted using the RiboPure™ RNA purification kit for yeast (Life technologies, Ambion). The quality and quantity of the RNA was assayed using a BioAnalyzer (Agilent, USA). About 2 ng of total RNA was used for sequencing based on Illumina HiSeq 2000 platform. Library construction was performed using the Illumina Trueseq kit at BGI Tech, Hong Kong.

### RNA-seq data analysis

The generated pair-end reads (FASTQ format) were checked for their quality using the FastQC tool. The files were groomed to meet the Sanger-scaled quality values with ASCII offset 33. The groomed paired-end reads were then mapped to the reference genome of *Y. lipolytica CLIB122 GCA_000002525* [51] using Tophat2 [52]. The gene and transcript expression levels were estimated and normalized based on FPKM (fragments per kilobase of exon per million fragments mapped) which were estimated using Cufflinks [53] with standard parameters and a *p* value cut-off set to 0.05. Gene ontology (GO) analysis was performed by using Blast2GO programs [54–56]. All raw sequence reads were deposited into the NCBI Sequence Read under the accession number of GSE83109.

## Additional file

**Additional file 1. Figure S1.** Biomass, products formation and substrate consumption in a pO_2_ uncontrolled culture of *Y. lipolytica* grown on a mixture of glycerol and spruce biomass hydrolysate. **Figure S2.** Gene ontology analysis of differentially expressed genes compared between pO_2_ controlled before vs. after glycerol limitation. **Figure S3.** Gene ontology analysis of differentially expressed genes compared between pO_2_ uncontrolled before vs. after glycerol limitation. **Figure S4.** Significant pathways and enzymes in the central metabolism of *Y. lipolytica* showing the differential gene expression between cell samples taken at pO_2_ controlled conditions before and after glycerol limitation (arrow). **Figure S5.** Flux distribution of *Y. lipolytica* grown on glycerol and glucose with pO_2_ control. Values are in mmol g_DCW_^−1^ h^−1^ normalized to glycerol uptake rate which is set as 100%. Arrows indicated flux towards biomass precursors.
